# Immune Gene Signature Delineates a Subclass of Papillary Thyroid Cancer with Unfavorable Clinical Outcomes

**DOI:** 10.3390/cancers10120494

**Published:** 2018-12-05

**Authors:** Kyuryung Kim, Sora Jeon, Tae-Min Kim, Chan Kwon Jung

**Affiliations:** 1 Cancer Research Institute, College of Medicine, The Catholic University of Korea, Seoul 06591, Korea; kyuryung.kim@catholic.ac.kr (K.K.); thfk38@nate.com (S.J.); 2 Department of Medical Informatics, College of Medicine, The Catholic University of Korea, Seoul 06591, Korea; 3 Department of Biomedicine & Health Sciences, Graduate School, The Catholic University of Korea, Seoul 06591, Korea; 4 Department of Hospital Pathology, College of Medicine, The Catholic University of Korea, Seoul 06591, Korea

**Keywords:** papillary thyroid carcinoma, immunity, molecular taxonomy, non-negative matrix factorization, survival

## Abstract

Papillary thyroid carcinoma (PTC) represents a heterogeneous disease with diverse clinical outcomes highlighting a need to identify robust biomarkers with clinical relevance. We applied non-negative matrix factorization-based deconvolution to publicly available gene expression profiles of thyroid cancers in the Cancer Genome Atlas (TCGA) consortium. Among three metagene signatures identified, two signatures were enriched in canonical *BRAF*-like and *RAS*-like thyroid cancers with up-regulation of genes involved in oxidative phosphorylation and cell adhesions, respectively. The third metagene signature representing up-regulation of immune-related genes further segregated *BRAF*-like and *RAS*-like PTCs into their respective subgroups of immunoreactive (IR) and immunodeficient (ID), respectively. *BRAF*-IR PTCs showed enrichment of tumor infiltrating immune cells, tall cell variant PTC, and shorter recurrence-free survival compared to *BRAF*-ID PTCs. *RAS*-IR and *RAS*-ID PTC subtypes included majority of normal thyroid tissues and follicular variant PTC, respectively. Immunopathological features of PTC subtypes such as immune cell fraction, repertoire of T cell receptors, cytolytic activity, and expression level of immune checkpoints such as and PD-L1 and CTLA-4 were consistently observed in two different cohorts. Taken together, an immune-related metagene signature can classify PTCs into four molecular subtypes, featuring the distinct histologic type, genetic and transcriptional alterations, and potential clinical significance.

## 1. Introduction

The incidence of thyroid cancer has been rapidly increasing worldwide, especially in Korea (15 times of increase) over the past few decades [1]. These trends are mainly driven by an increase in the detection of papillary thyroid carcinoma (PTC) which represents more than 80 percent of all thyroid cancers [1,2]. In the Unites States, the overall incidence rate increased by an average of 3% annually between 1974 and 2013 [2]. The American Cancer Society has estimated that numbers of new cases and deaths from thyroid cancer in the Unites States for 2018 will be 53,990 (40,900 in women and 13,090 in men) and 2060 (1100 women and 960 men), respectively (The American Cancer Society; www.cancer.org). Most patients with PTC have excellent prognosis after surgery. They are more likely to die from other diseases. However, recurrence and death can occur more than 30 years after initial diagnosis of PTC [3].

PTC is a heterogeneous disease characterized by more than 10 histologic variants with disparate molecular phenotypes and clinical behaviors [4]. Microscopic variants with more aggressive clinical outcomes than classic PTC include tall cell, columnar cell, and hobnail variants [5]. Other variants of PTC associated with a less favorable prognosis include the solid variant and diffuse sclerosing variant, although controversy remains [5]. In contrast, encapsulated variant of PTC has an excellent prognosis. It can achieve 100% survival rate, although the tumor may develop regional nodal or distant metastasis [4]. Most tumors previously known as non-invasive encapsulated follicular variant of PTC (EFVPTC) are now reclassified as a noninvasive follicular thyroid neoplasm with papillary-like nuclear features (NIFTP), which is not cancer but can be considered a borderline tumor with uncertain malignant potential [4].

Recent advances in next-generation sequencing based cancer genomic research have explored mutational and transcriptional landscape of PTCs. The Cancer Genome Atlas (TCGA) study of PTC has demonstrated that *BRAF*-like and *RAS*-like PTCs significantly differ in their genomic, transcriptomic, epigenomic, and proteomic profiles [6]. *BRAF*-like PTCs have classical papillary morphology and high levels of mitogen-activated protein kinase (MAPK) pathway signaling whereas *RAS*-like PTCs show a follicular growth pattern and low levels of MAPK pathway signaling [6]. *BRAF*-like PTCs are more clinically and molecularly heterogeneous than *RAS*-like tumors. In the initial analysis of the TCGA dataset, data on first recurrence were unavailable. The risk of recurrence was evaluated using the American Thyroid Association (ATA) risk stratification system [5] and metastasis, age, completeness of resection, invasion, and size (MACIS) system from the Mayo Clinic [7]. A study of RNA sequencing has observed that about 10% of all genes differentially expressed between PTCs with or without *BRAF* V600E are related to immune function pathways [8]. PTCs with *BRAF* V600E mutation have lower levels of immune/inflammation function gene expression and lymphocyte infiltration than *BRAF*-wild type PTCs. Another study using RNA sequencing data of TCGA has found that increased immune cell enrichment scores in PTCs are associated with low thyroid differentiation score and *BRAF* V600E mutation while the expression of immunosuppressive markers is higher in *BRAF* V600E positive PTCs [9]. These findings suggest that immune signature might have prognostic value in patients with PTC. In addition, multiple lines of evidence indicate that the coexistence of *BRAF* V600E and *TERT* promoter mutations is associated with aggressive clinical behavior and poor clinical outcome in PTC patients [10,11,12]. However, the potential relationship among the immune signatures, somatic mutations and patient prognosis in PTC is largely unknown.

A number of gene expression-based algorithms have been proposed for latent feature selection or deconvolution assuming that bulk-level sequencing of primary tumors represents an admixture of heterogeneous cell populations. As a technique of blind source separation [13], non-negative matrix factorization (NMF) can identify a small number of ‘metagene signatures’ from a gene expression profile that can be summarized in terms of metagene signatures [14]. NMF has been used for cancer gene expression profiles to infer the abundance of stromal components [15] and tumor classification based on immune cell abundance [16]. Along with NMF, other algorithms have also been proposed for direct deconvolution of tumor cell admixtures using a prior information such as cell type-specific expression profiles or gene members. For example, CIBERSORT implements a linear support vector regression to infer the relative abundance of 22 immune cell subsets in tumor expression profiles [17]. Similar algorithms using sets of immune genes representing various immunological contexts have facilitated immunoprofiling of multiple cancer types [18].

In the present study, we obtained a large-scale gene expression profile of PTC including tumor-adjacent normal thyroid tissue from TCGA consortium [6]. We applied NMF for thyroid expression profiles to identify three metagene signatures, one of which represented up-regulation of immune-related genes. We observed that this signature could refine the previously proposed two molecular PTC classes—*BRAF*-like and *RAS*-like PTCs. These identified PTC clusters were compared with previously proposed multiomics-based PTC clusters. They were also evaluated for immunologic features including immune cell abundance estimated by CIBERSORT algorithm. Of note, we evaluated the clinical utility of PTC clusters by correlative analyses with clinicopathological features including recurrence-free survival. Our observed findings were largely consistent across independent cohorts of our PTC expression profiles and a public one [19].

## 2. Results

### 2.1. Deconvolution of PTC Expression Profiles into Key Metagene Signatures

**Figure 1:**
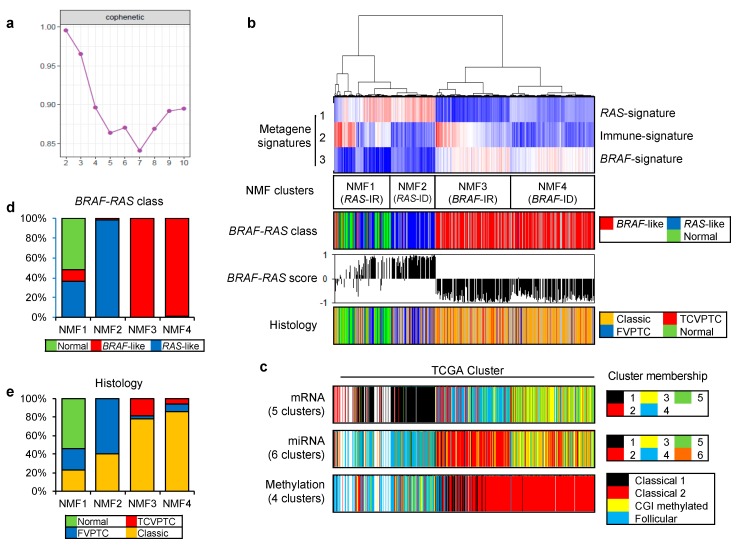
Gene expression profiles and non-negative matrix factorization (NMF)-driven metagene signatures of papillary thyroid carcinoma (PTC) in TCGA cohort. (**a**) Cophenetic correlation coefficients (*y*-axis) plotted against the number of metagene signatures (2 to 10; *x*-axis). A drop in stability was marked between 3 and 4 metagene signatures, suggesting that at least three metagene signatures were present in TCGA PTC expression profiles; (**b**) TCGA PTC metagene expression profiles clustered according to levels of three metagene signatures (Metagene signatures 1–3; *left*, annotated as ‘*RAS*-’, ‘Immune-’ and ‘*BRAF*-signatures’, respectively) revealing four NMF clusters (NMF1–NMF4 corresponding to *RAS*-IR, *RAS*-ID, *BRAF*-IR, and *BRAF*-ID, respectively). In the heatmap, red and blue represent increased and decreased levels of metagene signatures, respectively. Also shown are *BRAF-RAS* classes and scores of individual PTCs available in TCGA consortium. Three PTC histological types are shown with normal thyroid epithelium; (**c**) Three PTC clustering schemes with respect to mRNA, miRNA, and DNA promoter methylation are shown as proposed by TCGA consortium; (**d**) Bar plots showing the proportion of *BRAF*-like and *RAS*-like PTC with normal thyroid across four NMF clusters; (**e**) PTC histology in four NMF clusters. TCVPTC = tall cell variant of PTC; FVPTC = follicular variant of PTC.

To identify key metagene signatures that could explain heterogeneous PTC gene expression profiles in reduced dimensions, we performed NMF clustering of 568 RNAseq-based gene expression profiles (501 PTCs with 8 matched metastatic and 59 matched tumor-adjacent non-tumors) available in TCGA consortium [6]. A plot of cophenetic correlation coefficients, a measure of stability across the number of metagenes examined, showed that at least three metagene signatures were present in the expression profiles, as shown in Figure 1a.

Figure 1b. shows that 568 gene expression profiles of PTC and thyroid normal samples are segregated into four NMF clusters (NMF1–NMF4) based on the level of three metagene signatures. When we compared these NMF clusters with TCGA-based *BRAF*-*RAS* classes annotated according to the presence of driver mutations of *BRAF* and *RAS* genes, metagene signatures 1 and 3 were mostly enriched in *RAS*-like and *BRAF*-like PTC classes, respectively, as shown in Figure 1b. Thus, we annotated metagene signatures 1 and 3 as ‘*RAS*-signature’ and ‘*BRAF*-signature’, respectively. *RAS*-like and *BRAF*-like PTCs have been previously proposed as two molecular subtypes of PTC including a majority of histologic classes of predominantly follicular growth pattern and papillary growth pattern (classical PTC and tall cell variant [TCV] of PTC), respectively [6]. In addition, we also observed that normal thyroid expression profiles were clustered along with *RAS*-like PTCs, as shown in Figure 1b,d. Of note, metagene signature 2 further segregated *RAS*-like and *BRAF*-like PTCs into their respective two subgroups. In the case of *RAS*-like subtypes, metagene signature 2 was enriched in NMF cluster 1 that included all normal thyroid tissues while NMF cluster 2 was relatively enriched with follicular variant of PTC (FVPTC), as shown in Figure 1b,e. Metagene signature 2 also segregated *BRAF*-like PTCs: NMF cluster 3 PTCs were more enriched with TCVPTC than NMF cluster 4.

The clustering of PTC based on multiomics data such as mRNA, miRNA, and DNA promoter methylation has proposed the presence of multiple PTC clusters [6]. Thus, we compared our four NMF clusters with previously proposed multiomics-based clusters, as shown in Figure 1c. The split of NMF clusters 3 and 4 was similarly observed with that of mRNA cluster 3 and 4 by five-mRNA clustering and miRNA clusters 2 and 3 by six-miRNA clustering proposed by TCGA consortium. In addition, clusters annotated as ‘classical 1’ and ‘classical 2’ by the four-DNA methylation-based clustering were both assigned to *BRAF*-like PTCs with enrichment of metagene signature 3/*BRAF*-signature. Those belonging to ‘classical 2’ cluster showed an enrichment of metagene signature 2. Although the presence of multiple PTC clusters has been previously proposed, multiomics data-driven PTC clusters have not been properly evaluated and a functional interpretation of metagene signature 2 is largely unknown.

### 2.2. Functional Annotation of Immune-Related Metagene Signature

To functionally interpret metagene signatures, we performed pre-ranked gene set enrichment analysis (GSEA) [20]. High-ranked genes in three metagene signatures were enriched in molecular functions representing oxidative phosphorylation, cellular immunity, and ribosome/cell adhesions, respectively, as shown in Supplementary Table S1. It has been previously reported that *RAS*-like and *BRAF*-like thyroid cancers can activate the metabolic pathway and cell adhesion molecule/extracellular matrix receptor interaction pathways, respectively [19], consistent with our observation. GSEA also revealed that high-ranked genes in metagene signature 2 were largely associated with immune-related functional gene sets such as those associated with chemokine/cytokine secretions and leukocyte behaviors. Top ranked genes in metagene signature 2 were *CCL21* and *CCL19* encoding C-C motif chemokine ligands 21 and 19 precursors, respectively, as shown in Supplementary Table S2. Based on these results, we annotated metagene signature 2 as ‘Immune-signature’, as shown in Figure 1b. Immunoreactive (IR) and immunodeficient (ID) subgroups were distinguished by higher and lower levels of metagene signature 2/immune-signature, respectively. Therefore, we further annotated NMF clusters 1, 2, 3, and 4 as *RAS*-IR, *RAS*-ID, *BRAF*-IR, and *BRAF*-ID, respectively.

**Figure 2:**
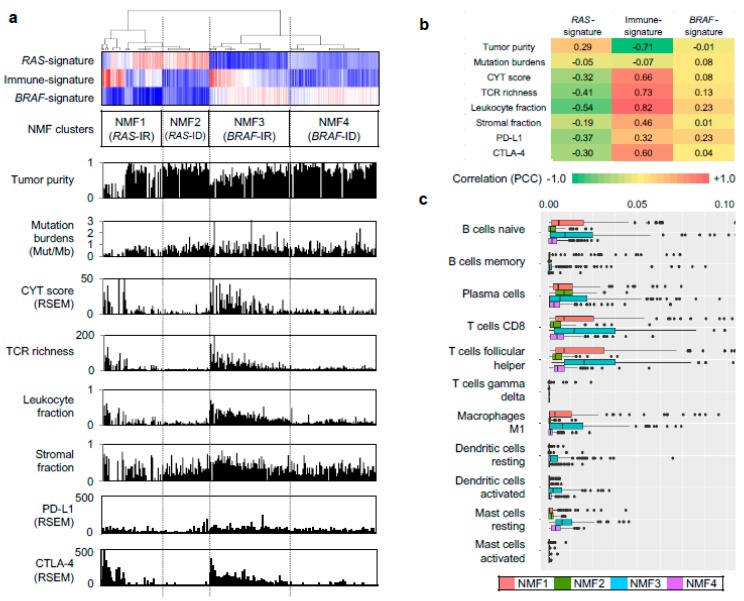
Characterization of immune signature. (**a**) Three metagene signatures and four NMF clusters are shown as Figure 1c. Eight immune-related genomic and pathologic features shown are tumor purity, mutation burden, CYT score, TCR richness, fraction of leukocytes and stromal cells, and expression level of PD-L1, CTLA-4 immune checkpoints; (**b**) Pearson correlation coefficients calculated for possible pairs of three metagene signature levels and eight immune-relate features across TCGA PTC expression profiles. A heatmap shows the level of correlation with a color legend; (**c**) Immune cell abundance (*x*-axis) estimated by CIBERSORT algorithm for 11 immune cell subsets (*y*-axis). Among 22 subsets of CIBERSORT output (LM22), those that are significant (*p* < 0.05; ANOVA) against the four NMF clusters are shown.

We further evaluated other genomic-pathologic features associated with immune cell abundance such as tumor purity [21], total mutation burden, CYT activity score (i.e., the geometric mean of the expression of PRF1 and GZMA) [22], TCR richness (i.e., the number of T-cell clones with unique TCRs) [18], expression-based estimates of immune cells and stromal cells [23], and expression levels of immune checkpoints of PD-L1 and CTLA-4, as shown in Figure 2a. Correlation levels were also measured between three metagene signatures and immune-related features, as shown in Figure 2b. In the case of immune-signature (metagene signature 2), four immune-related features (leukocyte fraction, TCR richness, CYT score, and the expression level of CTLA-4) were substantially correlated with the extent of enrichment for metagene signature (*r* = 0.82, 0.73, 0.66, and 0.60, respectively), as shown in Figure 2b. These features were also highly elevated in NMF cluster 3/*BRAF*-IR while they were suppressed in NMF cluster 2/*RAS*-ID and 4/*BRAF*-ID, as shown in Figure 2a. The inverse correlation of immune-signature with tumor purity (*r* = −0.71) is likely due to the relationship between leukocyte fraction and tumor purity [21].

**Figure 3:**
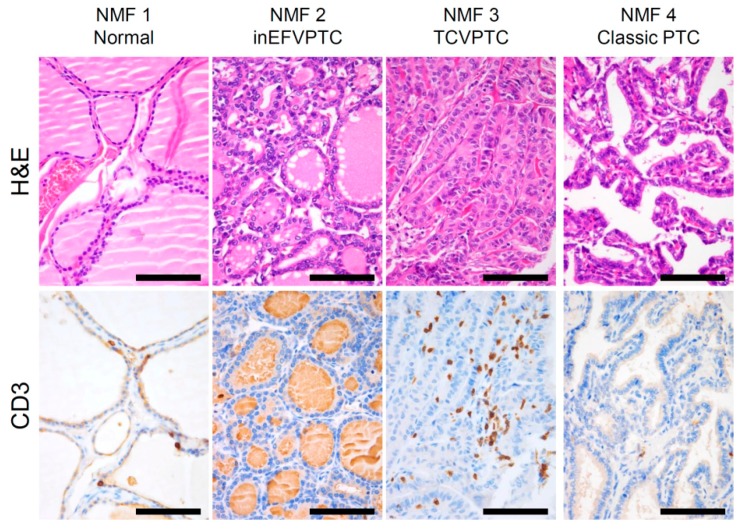
H&E staining of papillary thyroid carcinoma and adjacent normal thyroid tissue across the 4 NMF clusters with representative images showing staining for pan T-cell marker CD3. inEFVPTC = invasive encapsulated follicular variant of papillary thyroid carcinoma; TCVPTC = tall cell variant of PTC. Scale bar = 100 μm.

We further explored which immune cell subsets were differently enriched across four NMF clusters by using the CIBERSORT algorithm [17]. Figure 2c. shows estimated immune cell abundance for 11 immune cell types across four NMF clusters. Among 22 immune subsets, significantly differential enrichments across four NMF clusters are shown (*p* < 0.05; ANOVA). Various immune cell subtypes including B cells, T cells, macrophage M1, dendritic cells, and mast cells showed differential enrichments. The majority of them were enriched in NMF clusters one and three, consistent with the enrichment of immune-signature (metagene signature 2). These findings support that levels of metagene signature 2/immune-signature are associated with immune activity or the abundance of tumor infiltrating immune cells. The representative histologic images from each cluster are shown in Figure 3.

### 2.3. Prognostic Impact of NMF Clustering-Based PTC Classification in the TCGA Dataset

Baseline characteristics of NMF clustering subgroups of patients at initial surgery are shown in Table 1. No significant difference in age, sex, or initial distant metastases was found among the four groups. NMF clusters three and four had more frequent TCVPTC, extrathyroidal extension, advanced pT stage, lymph node metastasis, advanced AJCC stage, *BRAF*-like molecular phenotype, and high risk of recurrence. In subgroup analysis between NMF clusters three and four, cluster three patients had significantly higher rate of TCVPTC, extrathyroid extension, thyroiditis, and advanced tumor stage.

In univariate logistic regression analysis, as shown in Table 2, factors significantly associated with the status of disease recurrence were age of ≥55 years (*p* = 0.032), extrathyroidal extension (*p* = 0.008), pT3-4 stage (*p* = 0.007), lymph node metastasis (*p* = 0.003), high burden of nonsynonymous mutations (*p* = 0.020), and NMF cluster 3 (*p* = 0.008). In multivariate analysis, as shown in Table 2, lymph node metastasis (*p* = 0.001), high burden of nonsynonymous mutations (*p* = 0.021), and NMF cluster 3 (*p* = 0.010) remained significant factors associated with the status of disease recurrence.

**Figure 4:**
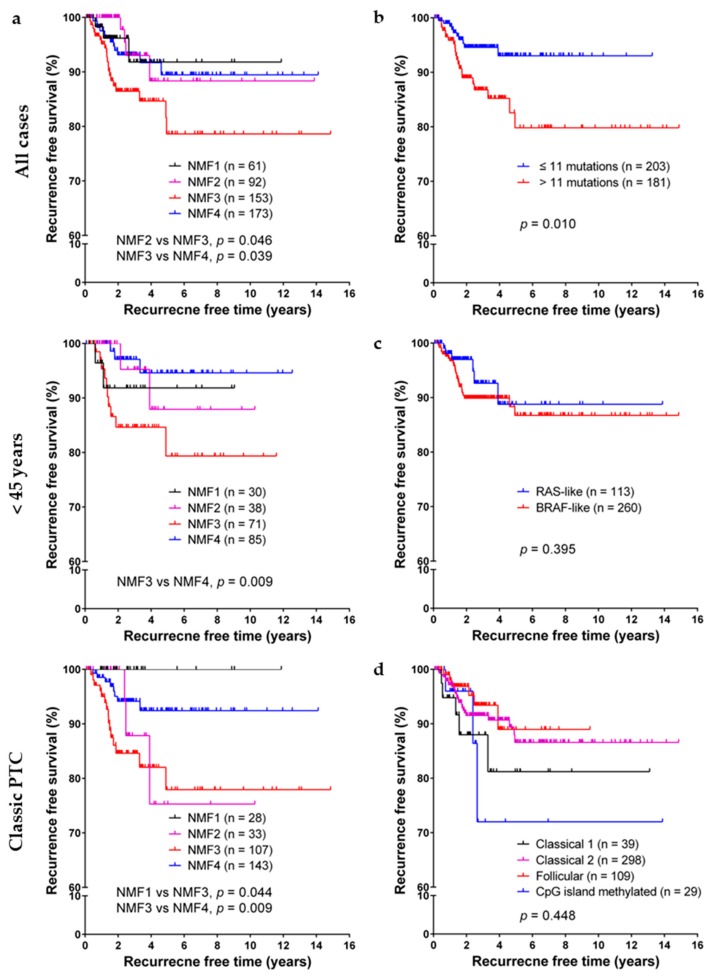
Survival analysis. (**a**) Kaplan–Meier survival curves of papillary thyroid carcinoma (PTC) patients on disease recurrence free survival stratified according to the four NMF clusters; (**b**) Burden of nonsynonymous mutations; (**c**) *RAS-BRAF* PTC classes; and (**d**) DNA methylation clusters.

In Kaplan–Meier analyses of PTC recurrence-free probability, as shown in Figure 4, NMF cluster three was significantly associated with lower recurrence free survival rate, especially in subgroups of patients less than 45 years of age and patients with classic PTC. Old age (≥55 years), extrathyroidal extension, lymph node metastasis, and high burden of nonsynonymous mutations were also associated with short recurrence free survival, as shown in Table 3. However, no significant difference was found among subgroups classified by dichotomous *RAS*-like/*BRAF*-like clustering, mRNA clusters (clusters 1–5), microRNA clustering (clusters 1–6), or DNA methylation clustering (follicular, GpG methylated, classical 1, and classical 2 clusters). In multivariate Cox regression analysis, lymph node metastasis, high burden of nonsynonymous mutations, NMF cluster three had a negative influence on recurrence free survival, as shown in Table 3.

### 2.4. Validation of NMF Clustering-Based Classification in Two Different Cohorts

**Figure 5:**
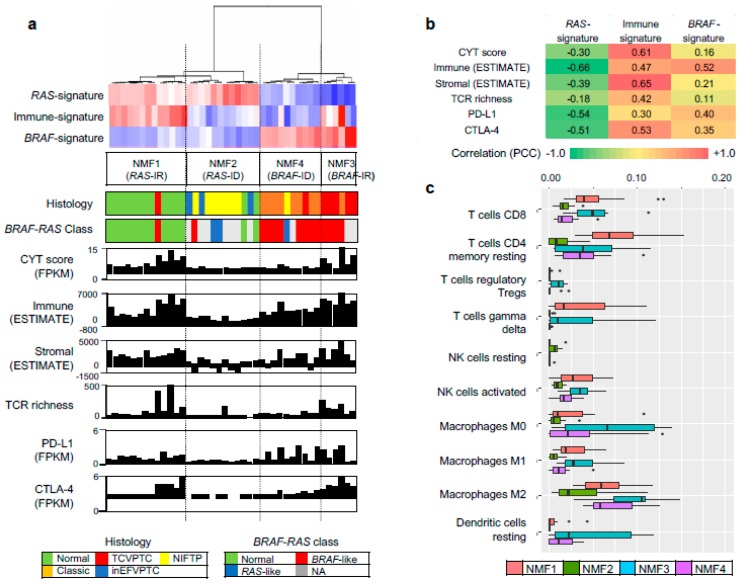
NMF clusters and immune features of CMC cohort. (**a**) Four NMF clusters determined by metagene projection of three metagene signatures for 41 thyroid profiles of CMC cohort. Five histological types and mutation-based *BRAF*-/*RAS*-like classes are shown with corresponding legends below. Six immune-related features are shown, including CYT score, immune/stromal ESTIMATE scores, TCR richness, and expression levels of PD-L1 and CTLA-4; (**b**) A heatmap showing correlation levels between three metagene signatures and six immune features; (**c**) Abundance of 10 immune cell subsets (*p* < 0.05; ANOVA test) with respect to four NMF clusters.

**Figure 6:**
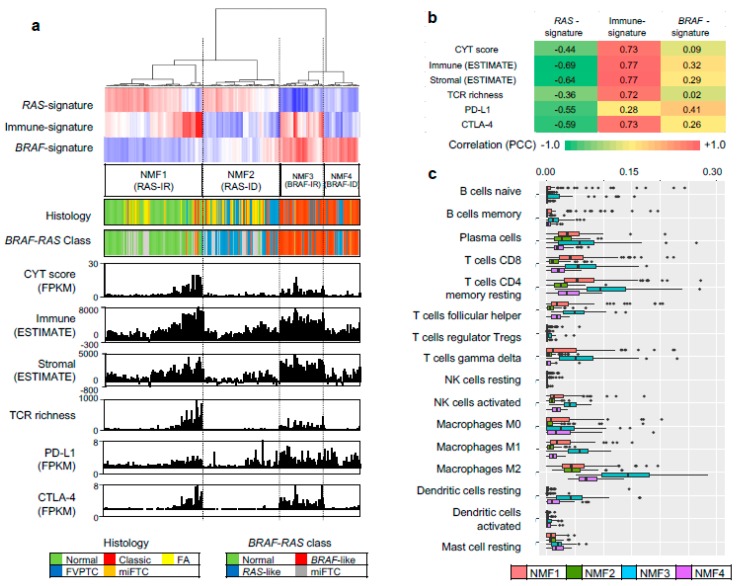
NMF clusters and immune features of SNU cohort. (**a**) Four NMF clusters for 261 thyroid expression profiles in SNU cohort. Histological subtypes, mutation-based *BRAF-RAS* classes, and six immune features are also shown; (**b**) Correlation between signature levels and immune features; (**c**) Abundance of 16 immune cell subsets.

**Figure 7:**
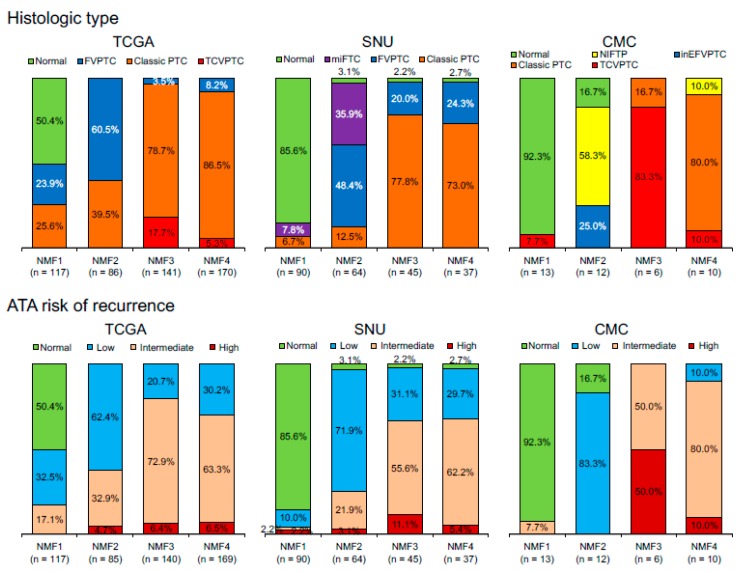
Distribution of histologic types and American Thyroid Association (ATA) risk of recurrence with respect to four NMF clusters in three different datasets. TCGA cohort includes normal thyroid and follicular, classic, and tall cell variants of PTC. SNU cohort includes normal, minimally invasive follicular thyroid carcinoma (miFTC), and follicular variant of PTC (FVPTC) and classic PTC. CMC cohort includes normal, non-invasive follicular thyroid neoplasm with papillary-like nuclear features (NIFTP), and invasive encapsulated follicular (inEFV), classic, and tall cell variants (TCV) of PTC. NIFTP was incorporated into the ATA low risk group.

To evaluate three metagene signature-based classification of PTC in independent gene expression datasets, we performed RNAseq for 27 thyroid tumors and 14 tumor-adjacent normal thyroid tissues. A total of 41 thyroid expression profiles (CMC cohort) were subjected to metagene projection with three metagene signatures (*RAS*-, *BRAF*- and immune-signatures) to yield four NMF clusters with a similar enrichment pattern of metagene signatures and the association with immune-histological presentations, as shown in Figure 5a. For example, NMF clusters 1/*RAS*-IR and 3/*BRAF*-IR included the majority of normal thyroid tissue and TCVPTC, respectively. The level of immune-related features such as CYT, immune scores from ESTIMATE algorithm, TCR richness, and the expression level of CTLA-4 were highly elevated in NMF3/*BRAF*-IR PTCs. Correlation coefficients were also high with immune signature, as shown in Figure 5b. Ten immune cell subsets (*p* < 0.05; ANOVA) were illustrated, demonstrating that T cells and macrophages were highly infiltrated in tumors belonging to IR clusters (NMF3 and NMF1) compared to those belonging to ID clusters (NMF2 and NMF4), as shown in Figure 5c. We also performed similar analysis for another public SNU cohort of 261 thyroid expression profiles. The presence of four NMF clusters whose enrichment pattern with histology and immune-related features was similarly observed with TCGA and CMC cohort, as shown in Figure 6. For example, CYT score, immune/stromal ESTIMATE scores, TCR richness, and the expression of CTLA-4 showed high levels of correlation (*r* > 0.7) with the level of signature 2 in SNU cohort. These findings suggest that our three metagene signature-based PTC classification is consistent across a number of PTC expression profiles. In addition, the relationship between NMF clusters, histologic type, and ATA recurrence risk were investigated across three cohorts, as shown in Figure 7. The SNU cohort included minimally invasive follicular thyroid carcinomas (miFTCs) that were mostly classified into NMF cluster two. In the CMC cohort, most NIFTPs were classified into NMF cluster two. In all three cohorts, normal thyroid and ATA low risk groups were mostly classified into NMF clusters one and two, respectively.

## 3. Discussion

We identified clinically relevant metagene signatures that could classify PTCs into four groups and predict disease recurrence after initial treatment. The binomial classification of PTCs into *RAS*-like and *BRAF*-like tumors was further divided into NMF1 (*RAS*-IR), NMF2 (*RAS*-ID), NMF3 (*BRAF*-IR), and NMF4 (*BRAF*-ID). Normal thyroid tissue was enriched in NMF1. NMF3 showed *BRAF*-like molecular features and enrichment of tumor infiltrating immune cells. The level of immune-signature was suppressed in NMF clusters two and four. NMF3 was an independent prognostic marker for disease recurrence in TCGA cohort. We confirmed that immunopathological features and NMF classification of PTC developed from TCGA cohort were consistently replicated in SNU and CMC cohorts. Interestingly, NMF2 was enriched with miFTC and NIFTP in SNU and CMC cohorts, respectively. 

Robust molecular classification of thyroid tumors with clinical implication is challenging. MAPK pathway is activated in approximately 70% of PTCs mainly by *BRAF* V600E and *RAS* activating mutations [24]. The predominance of *BRAF* and *RAS* mutations in PTCs and their mutually exclusivity have led to the discovery of two molecular subtypes of PTC–*BRAF*-like and *RAS*-like PTCs [24,25]. Although TCGA consortium has recently confirmed the presence of these two molecular PTC subtypes and that they may be better correlated with molecular signaling and tumor differentiation than histologic subtypes [6], we observed that *BRAF*-like and *RAS*-like PTCs had similar recurrence-free survival, as shown in Figure 4. Although the TCGA report has proposed that additional molecular PTC subtypes based on multiomics analyses may be present with distinct molecular pathways involved, their clinical implication is still largely unknown.

NMF-based deconvolution technique has been proven to be useful in the decomposition of multiple cellular composition given that a bulk-level tumor transcriptome is a heterogeneous cellular admixture of tumor cells and tumor-infiltrating non-tumor cells such as immune and stromal cells [15]. When applying NMF for PTC gene expression profiles of TCGA dataset, we employed a stability measure (i.e., cophenetic correlation). The highest correlation was observed for clusters two and three, suggesting that at least three metagene signatures are present in the TCGA PTC dataset, as shown in Figure 1a. It is conceivable that two of three metagene signatures correspond to *BRAF*- and *RAS*-like PTC subtypes. We noted that the remaining metagene signature was enriched with immune-related genes, suggesting that expression-level immune activity could be an additional feature in PTC categorization. Among the four PTC clusters, *BRAF*-IR/NMF3 cluster demarcated a subgroup of *BRAF*-like PTC with unfavorable prognosis, as shown in Figure 4a, and distinct immune-related features compared to *BRAF*-ID PTCs, as shown in Figure 2. The metagene signature-based PTC clusters and their immunologic features were consistently observed across three PTC expression cohorts (TCGA, CMC and SNU). For broad applicability, expression levels of *RAS*-, *BRAF*- and immune-metagene signatures that can be projected onto microarray- or RNAseq-based PTC expression profiles are provided, as shown in Supplementary Table S2.

The recent success of immune checkpoint blockade treatment in various types of solid tumors [26,27] with potent and durable response has suggested its potential use for refractory, advanced thyroid cancers [28]. Currently, total mutation burdens [29] and expression levels of checkpoint inhibitors (e.g., PD-L1 levels for anti-PD1-PD-L1 treatments) have been proposed as predictors of clinical response [30]. In our study, somatic mutation burdens were not substantially different across NMF PTC clusters, as shown in Figure 2a. Of note, we observed that CTLA-4 and PD-L1 expression was relatively up-regulated in *BRAF*-IR compared to that in *BRAF*-ID PTCs, as shown in Figure 2a. Therefore, patients with *BRAF*-IR PTCs may be candidates for PD1, PD-L1, or CTLA-4 blockade therapy. Along with unfavorable clinical outcomes of *BRAF*-IR PTCs, their potential eligibility to immune checkpoint blockade treatment should be investigated further.

Some studies have reported gene expression-based immunoprofiling of PTC using TCGA data. Na and Choi have employed gene expression-based PTC differentiation and immune scores [9]. They observed that high immune score was associated with *BRAF* V600E mutation, low thyroid differentiation score, high expression of immunosuppressive markers (PD-L1, CTLA-4, and HLA-G), and shorter recurrence-free survival [9]. Kuo et al. [31] have divided PTCs in the TCGA cohort into two groups with lymphocyte infiltration < 1% and ≥ 1%. PTCs with lymphocyte infiltration ≥ 1% had higher rates of classic histology, multifocality, and lymph node metastasis than those with lymphocyte infiltration < 1%. Gene expression profiles for the group with infiltrating lymphocytes ≥ 1% were enriched with genes related to hematopoiesis, cytokine production, and cell adhesion molecules as well as immune-related pathway [31]. However, there was no significant difference in recurrence-free survival rate, *BRAF* mutation status, or expression of PD-L1 between the two groups. In the present study, expression levels of CTLA-4 and PD-L1 were up-regulated in *BRAF*-IR group with tumor infiltrating immune cells. The *BRAF*-IR group had the highest recurrence rate.

Our analysis on the abundance of individual immune cell subsets showed overall enrichment of immune cells in *BRAF*-like and *RAS*-like PTCs instead of specific immune cell types. Across three cohorts examined, T lymphocytes and myeloid cells such as macrophages and dendritic cells were commonly overrepresented in IR PTCs. In the case of T cells, CD8+ lymphocyte infiltration within tumor cells has been generally considered as an unfavorable prognostic feature across cancers including thyroid cancers [32]. Cunha et al. have found that enrichment with CD8+ tumor-infiltrating lymphocytes and COX2 expression are independent risk factors for disease recurrence of well differentiated thyroid cancer regardless of the concurrent presence of chronic lymphocytic thyroiditis [32]. When we further analyzed gene expression profiles of CD8+ T cells in the TCGA data, high *CD8* mRNA level was associated with lymph node metastasis (*p* = 0.008) and *BRAF*-like group (*p* < 0.001, data not shown). Concomitant up-regulation of CYT scores in *BRAF*-IR PTCs suggest that these tumors are enriched with cytolytic CD8+ T cells. Up-regulation of CTLA-4 and PD-L1 is suggestive of the exhaustion of infiltrated T cells and may explain unfavorable clinical outcomes of this PTC subtype. Adverse effects of regulatory T cells (Tregs) in antitumor immune response should also be considered since Tregs are enriched in *BRAF*-/*RAS*-IR PTCs [33]. Myeloid originating macrophages and dendritic cells are also enriched in *BRAF*-/*RAS*-IR PTCs. Tumor-associated macrophages (TAM) have been previously associated with clinical outcomes of PTC [34]. Further investigation is needed to determine the roles of different TAM subsets such as inflammatory phenotype 1 TAMs and suppressive phenotype 2 TAMs given their opposing roles in the tumor pathology [35].

## 4. Materials and Methods

### 4.1. Public PTC Transcriptome Data

We used two publicly available gene expression datasets. A total of 568 expression profiles for PTCs (*n* = 509) and tumor-adjacent normal thyroid tissues (*n* = 59) were obtained from TCGA consortium [6]. We downloaded RNAseq-based, gene-level normalized RSEM (RNA-Seq by Expectation Maximization) scores from Broad Firehose (https://gdac.broadinstitute.org). Among these 568 expression profiles, 501 were from primary PTC tumor tissues, 8 were matched metastatic tumors, and 59 were expression profiles of adjacent normal thyroid tissue. Clinicopathological information of these patients was obtained from TCGA data portal (https://portal.gdc.cancer.gov/) and the literature [6]. Tumor recurrence was defined as new biochemical or structural evidence of disease after initial surgical treatment.

In addition to the TCGA cohort, we obtained gene expression data for 180 thyroid tumors including 25 follicular adenomas, 30 FTCs, 48 FVPTCs, 77 PTCs, and matched 81 normal thyroid tissues from a public resource [19]. RNAseq FASTQ files were downloaded from European Nucleotide Archive database with corresponding accession numbers (http://www.ebi.ac.uk/data/view/PRJEB11591). We used FastQC and Trimmomatic [36] for quality check and trimming of these sequencing data, respectively. Splice-aware sequencing read alignment was done using TopHat2 [37]. Gene-level summary of expression levels into FPKM (fragments per kilobase million) was done using CuffLinks [38]. We called these 261 gene expression profiles obtained from 180 thyroid tumors and 81 normal tissues as a Seoul National University (SNU) cohort [19].

### 4.2. Transcriptome Sequencing and Data Processing

We performed RNAseq on an Illumina platform for 27 thyroid tumors and 14 matched normal specimens. The tumor set was composed of classical PTC (*n* = 9), TCVPTC (*n* = 7), invasive EFVPTC (*n* = 3), and NIFTP (*n* = 8). The enrollment of patients and the overall experimental process were approved by the Institutional Review Board of Seoul St. Mary’s Hospital, the Catholic University of Korea (KC16SISI0709). Histological examination and tumor purity check were done by a board-certified pathologist. Tissue RNAs were extracted and converted into cDNAs. Sequencing library was prepared according to the manufacturer’s instructions. Sequencing reads were generated using Illumina HiSeq2500 (Illumina, San Diego, CA, USA). Sequencing reads of FASTQ files were aligned and processed into gene-level expression profiles as described for SNU cohort. We describe the obtained 41 gene expression profiles as a Catholic Medical Center (CMC) cohort. The sequencing information of RNAseq is available in Supplementary Table S3.

### 4.3. NMF and Metagene Signatures of PTCs

NMF implemented in R packages (https://cran.r-project.org/package=NMF) was used to deconvolute log-transformed thyroid expression profiles of TCGA consortium. To determine the number of metagene signatures, we measured cophenetic correlation in a range of signature numbers (2 to 10 metagene signatures). The goal of NMF is to identify latent features in gene expression profiles by decomposing the original matrix into basis matrix or metagenes (hereafter, we will use ‘metagene signatures’) and metagene expression profiles [14]. To functionally annotate metagene signatures, we performed pre-ranked GSEA using gene-level weight values of individual metagene signatures [20]. For metagene signature-based clustering, we performed hierarchical clustering of metagene expression profile. The resulting clusters were matched to PTC histology and multiomics-based cluster membership available in TCGA consortium. To apply TCGA-driven metagene signature-based clustering to other expression dataset, we employed metagene projection [39]. For metagene projection, positive linear combination of metagene signatures obtained in the model dataset (TCGA dataset) were projected onto other datasets (CMC and SNU datasets) using the Moore-Penrose generalized pseudoinverse with ginv function of R MASS library [39].

### 4.4. Immunoprofiling

To infer the relative abundance of tumor infiltrating immune cells, we used CIBERSORT [17]. The default set (LM22) was used to estimate the relative abundance of 22 immune cell types in individual specimens across three PTC expression cohorts. Immune-related features of PTCs including the tumor purity, mutation burdens, diversity of T cell receptor repertoire (TCR richness), and leukocyte/stromal cell fraction in TCGA cohort were obtained from a literature [18]. Cytolytic (CYT) score representing the activity of immune cytolytic effectors was calculated as geometric means of expression of GZMA and PRF1 as previously described [22]. For SNU and CMC datasets, we used ESTIMATE R packages to estimate the score representing the proportion of immune and stromal cells [23]. To estimate the diversity of TCR repertoire in RNAseq datasets of SNU and CMC cohorts, we used miXCR package [40].

### 4.5. Immunohistochemistry

Immunohistochemistry for pan T-cell marker CD3 was performed on 4 µm-thick tissue sections of formalin-fixed paraffin-embedded blocks using an automated immunostaining system (GI100, Dako Omnis, Agilent Technologies, Santa Clara, CA, USA). Antigen retrieval was performed with high-pH EnVision FLEX Target Retrieval Solution (Agilent Technologies) for 30 min at 97 °C. Tissues sections were incubated with polyclonal rabbit anti-human CD3 antibody (1:100, Code No. A 0452, Agilent Technologies) for 20 min at room temperature, followed by visualization with EnVision FLEX visualization system (EnVision/HRP for 20 min and chromogen substrate for 5 min). The specimens were then counterstained with Hematoxylin for 3 min.

### 4.6. Statistical Analysis

Analysis of variance (ANOVA) was performed to compare means of gene expression values among groups. Relationships between clinicopathologic features and gene expression profiles were analyzed using parametric (chi-square test) and non-parametric (Fisher’s exact) assessments where appropriate. Univariate binomial logistic regression analysis of variables was performed to determine whether clinicopathologic variables and molecular clustering were significantly associated with tumor recurrence. Disease recurrence free survival curves were plotted using the Kaplan–Meier method. Statistical differences between survival curves were calculated using the log-rank test. For multivariate survival analysis of variables affecting disease-free survival, the Cox proportional-hazard model was used. All statistical values were calculated using Prism (version 6.05, GraphPad Software, La Jolla, CA, USA) and statistical software program SPSS (version 21.0, IBM Corp, Armonk, NY, USA). *p* values of less than 0.05 were considered to indicate statistically significant differences.

## 5. Conclusions

The immune-related metagene signature identified four clinically distinct subgroups of PTCs in the present study. The risk of recurrence of PTC after initial treatment was the highest in immune reactive *BRAF*-like PTCs. This new classification provides novel insights into our understanding of immune response in PTCs and clinical application of molecular classification for the treatment and management of this tumor.

## Figures and Tables

**Table 1:** Relationship between non-negative matrix factorization (NMF) clusters and clinicopathologic features of papillary thyroid carcinoma in TCGA dataset. Extrathyroidal extension includes minimal microscopic and gross invasion. Tumor stage was determined by the seventh edition of American Joint Cancer Committee cancer staging system. * *p*-value was analyzed between cluster three and cluster four. MACIS: Metastasis, patient Age, Completeness of resection, local Invasion, and tumor Size. ATA: American Thyroid Association.

Characteristic	*n*	Cluster 1	Cluster 2	Cluster 3	Cluster 4	*p*-Value	*p*-Value *
Age (years)	500						
<45	228	30 (13.2%)	40 (17.5%)	72 (31.6%)	86 (37.7%)	0.788	0.720
≥45	272	34 (12.5%)	57 (21.0%)	86 (31.6%)	95 (34.9%)		
<55	335	44 (13.1%)	57 (17.0%)	102 (30.4%)	132 (39.4%)	0.095	0.096
≥55	165	20 (12.1%)	40 (24.2%)	56 (33.9%)	49 (29.7%)		
Sex						0.965	0.828
Female	340	42 (12.4%)	63 (18.5%)	108 (31.8%)	127 (37.4%)		
Male	124	17 (13.7%)	24 (19.4%)	37 (29.8%)	46 (37.1%)		
Histologic variant	464					<0.001	0.008
Classic	322	30 (9.3%)	34 (10.6%)	111 (34.5%)	147 (45.7%)		
Follicular variant	99	28 (28.3%)	52 (52.5%)	5 (5.1%)	14 (14.1%)		
Tall cell variant	34	0 (0.0%)	0 (0.0%)	25 (73.5%)	9 (26.5%)		
Other	9	1 (11.1%)	1 (11.1%)	4 (44.4%)	3 (33.3%)		
Extrathyroidal extension	500					<0.001	0.009
Absent	367	58 (15.8%)	87 (23.7%)	92 (25.1%)	130 (35.4%)		
Present	133	6 (4.5%)	10 (7.5%)	66 (49.6%)	51 (38.3%)		
Pathologic T (pT) stage	462					<0.001	0.024
pT1	134	26 (19.4%)	28 (20.9%)	38 (28.4%)	42 (31.3%)		
pT2	157	18 (11.5%)	38 (24.2%)	34 (21.7%)	67 (42.7%)		
pT3	153	15 (9.8%)	20 (13.1%)	64 (41.8%)	54 (35.3%)		
pT4	18	0 (0.0%)	1 (5.6%)	8 (44.4%)	9 (50.0%)		
Pathologic N (pN) stage	500					<0.001	0.720
pN0/NX	294	52 (17.7%)	84 (28.6%)	72 (24.5%)	86 (29.3%)		
pN1	206	12 (5.8%)	13 (6.3%)	86 (41.7%)	95 (46.1%)		
Initial distant metastasis	500					0.470	1.000
Absent	492	64 (13.0%)	94 (19.1%)	156 (31.7%)	178 (36.2%)		
Present	8	0	3 (37.5%)	2 (25.0%)	3 (37.5%)		
Tumor stage	498					<0.001	0.020
stage 1	283	44 (15.5%)	52 (18.4%)	86 (30.4%)	101 (35.7%)		
stage 2	51	10 (19.6%)	19 (37.3%)	4 (7.8%)	18 (35.3%)		
stage 3	109	10 (9.2%)	18 (16.5%)	45 (41.3%)	36 (33.0%)		
stage 4	55	0 (0.0%)	7 (12.7%)	23 (41.8%)	25 (45.5%)		
Thyroiditis						0.004	0.002
Absent	372	45 (12.1%)	72 (19.4%)	106 (28.5%)	149 (40.1%)		
Present	70	14 (20.0%)	9 (12.9%)	31 (44.3%)	16 (22.9%)		
*RAS* and *BRAF* signature						<0.001	1.000
*RAS*-like	118	40 (33.9%)	77 (65.3%)	0	1 (0.8%)		
*BRAF*-like	272	14 (5.1%)	1 (0.4%)	122 (44.9%)	135 (49.5%)		
MACIS	442					0.318	0.050
<6	315	41 (13.0%)	58 (18.4%)	87 (27.6%)	129 (41.0%)		
6–7	63	9 (14.3%)	13 (20.6%)	25 (39.7%)	16 (25.4%)		
7–8	39	4 (10.3%)	7 (17.9%)	15 (38.5%)	13 (33.3%)		
>8	25	1 (4.0%)	4 (16.0%)	11 (44.0%)	9 (36.0%)		
ATA^5^ recurrence risk	452					<0.001	0.159
Low	171	38 (22.2%)	53 (31.0%)	29 (17.0%)	51 (29.8%)		
Intermediate	257	20 (7.8%)	28 (10.9%)	102 (39.7%)	107 (41.6%)		
High	24	0 (0.0%)	4 (16.7%)	9 (37.5%)	11 (45.8%)		
Recurrence free status	479					0.048	0.063
Disease free	440	58 (13.2%)	88 (20.0%)	133 (30.2%)	161 (36.6%)		
Recurrent	39	3 (7.7%)	4 (10.3%)	20 (51.3%)	12 (30.8%)		

**Table 2:** Univariate and multivariate logistic regression analyses for risk factors of disease recurrence in the papillary thyroid carcinoma TCGA dataset. Variables showing a tendency of association with recurrence (*p* < 0.25) in the univariate analysis were included in the multivariate model. CI: confidence interval.

Characteristic	No. of Recurrences	Univariate Analysis		Multivariate Analysis	
		Hazard Ratio (95% CI)	*p*-Value	Hazard Ratio (95% CI)	*p*-Value
Age (years)			0.032		0.180
<55	22/334 (6.6%)	1 (reference)		1 (reference)	
≥55	19/152 (12.5%)	2.03 (1.06–3.87)		1.91 (0.74–4.89)	
Sex			0.214		0.476
Female	24/331 (7.3%)	1 (reference)		1 (reference)	
Male	13/119 (10.9%)	1.57 (0.77–3.19)		1.41 (0.55–3.58)	
Histologic variant			0.062		0.752
Non-aggressive variant	31/414 (7.5%)	1 (reference)		1 (reference)	
Aggressive variant	6/36 (16.7%)	2.47 (0.96–6.39)		1.22 (0.36–4.08)	
Extrathyroidal extension			0.008		0.374
Absent	23/359 (6.4%)	1 (reference)		1 (reference)	
Present	18/127 (14.2%)	2.41 (1.26–4.64)		1.49 (0.62–3.57)	
Pathologic T (pT) stage			0.007		0.501
pT1-2	16/287 (5.6%)	1 (reference)		1 (reference)	
pT3-4	21/161 (13.0%)	2.54 (1.29–5.02)		0.62 (0.15–2.49)	
Pathologic N (pN) stage			0.003		
pN0/NX	15/287 (5.2%)	1 (reference)		1 (reference)	0.001
pN1	26/199 (13.1%)	2.73 (1.40–5.29)		5.27 (2.02–13.74)	
*RAS* and *BRAF* signature			0.206		0.548
*RAS*-like	6/113 (5.3%)	1 (reference)		1 (reference)	
*BRAF*-like	24/260 (9.2%)	1.81 (0.72–4.57)		0.65 (0.16–2.61)	
Burden of nonsynonymous mutations			0.020		0.021
≤11 mutations	10/203 (4.9%)	1 (reference)		1 (reference)	
>11 mutations	21/181 (11.6%)	2.53 (1.16–5.54)		2.78 (1.17–6.62)	
NMF cluster			0.008		0.010
Non-NMF cluster 3	19/326 (5.8%)	1 (reference)		1 (reference)	
NMF cluster 3	20/153 (13.1%)	2.43 (1.26–4.70)		3.01 (1.31–6.95)	

**Table 3:** Univariate and multivariate Cox regression analyses for recurrence free survival in the papillary thyroid carcinoma TCGA dataset. CI: confidence interval.

Variables	Univariate Analysis	Multivariate Analysis	
		Hazard Ratio (95% CI)	*p*-Value
Age ≥ 55 years	0.014	1.81 (0.75–4.38)	0.187
Male	0.129	1.33 (0.56–3.14)	0.521
Aggressive variant	0.028	1.39 (0.49–3.95)	0.531
Extrathyroidal extension	0.017	1.28 (0.56–2.91)	0.560
Lymph node metastasis	0.002	4.98 (2.00–12.38)	0.001
*BRAF*-like tumor	0.395	1.63 (0.45–5.99)	0.459
Nonsynonymous mutations > 11 mutations	0.010	2.68 (1.20–5.99)	0.016
NMF cluster 3	0.007	1.42 (1.10–1.84)	0.007

## Supplementary Materials

The following are available online at http://www.mdpi.com/2072-6694/10/12/494/s1, Table S1: GSEA of three metagene signatures, Table S2: Gene-level weights and metagene factors for GSEA of 3 metagene signatures., Table S3: Sequencing information of RNAseq (CMC cohort).
